# Blockade of Neutrophil’s Chemokine Receptors CXCR1/2 Abrogate Liver Damage in Acute-on-Chronic Liver Failure

**DOI:** 10.3389/fimmu.2017.00464

**Published:** 2017-04-24

**Authors:** Arshi Khanam, Nirupma Trehanpati, Peggy Riese, Archana Rastogi, Carlos Alberto Guzman, Shiv Kumar Sarin

**Affiliations:** ^1^Department of Molecular and Cellular Medicine, Institute of Liver and Biliary Sciences, New Delhi, India; ^2^Department of Vaccinology and Applied Microbiology, Helmholtz Centre for Infection Research, Braunschweig, Germany; ^3^Department of Histopathology, Institute of Liver and Biliary Sciences, New Delhi, India; ^4^Department of Hepatology, Institute of Liver and Biliary Sciences, New Delhi, India

**Keywords:** acute-on-chronic liver failure, chemokine receptors CXCR1 and CXCR2, neutrophils, cell death, SCH 527123 antagonist

## Abstract

**Background:**

Neutrophils serve as critical players in the pathogenesis of liver diseases. Chemokine receptors CXCR1 and CXCR2 are required for neutrophil chemotaxis to the site of inflammation/injury and are crucial in hepatic inflammatory response. However, key mechanism of neutrophil-mediated liver injury in acute-on-chronic liver failure (ACLF) remains highly elusive; which could be targeted for the development of new therapeutic interventions.

**Methods:**

To demonstrate the role of CXCR1/CXCR2-expressing neutrophils in hepatic injury, we investigated CXCR1/CXCR2 receptor expression in 17 hepatitis B virus-related ACLF patients in comparison to 42 chronic hepatitis B and 18 healthy controls. Mechanism of neutrophil-mediated cell death was analyzed by *in vitro* coculture assays and correlated with the patient data. In addition, to find out any etiological-based variations in ACLF, 19 alcohol-related ACLF patients were also included.

**Results:**

In ACLF, neutrophils have high expression of CXCR1/CXCR2 receptors, which potentially participate in hepatocyte death through early apoptosis and necrosis in contact-dependent and -independent mechanisms. Importantly, blockade of CXCR1/CXCR2 with SCH 527123 antagonist significantly reduced cell death by targeting both the mechanisms. No etiology-based differences were seen between ACLF groups. Importantly, absolute neutrophil count was particularly higher in clinically severe ACLF patients and non-survivors (*p* < 0.0001). Multivariate analysis demonstrated ANC and CXCL8/IL-8 as a predictor of mortality. Further, receiver operating characteristics curve confirmed the cutoff of ANC >73.5% (sensitivity: 76.5% and specificity: 76.5%) and CXCL8/IL-8 >27% (sensitivity: 70% and specificity: 73%) in prediction of mortality.

**Conclusion:**

Blockade of CXCR1/CXCR2 diminished the production of inflammatory mediators and reduced cell death; therefore, pharmacological neutralization of CXCR1/CXCR2 could provide novel therapeutic target in the management of ACLF.

## Introduction

Acute-on-chronic liver failure (ACLF) is a distinct syndrome characterized by a rapid worsening of underlying chronic liver disease due to an acute insult, culminating in organ failure and high mortality (1). Liver transplantation remains potential therapeutic option for the patients with failed standard medical treatment and supportive measures (2). A better understanding of pathogenic mechanisms underlying development of ACLF is the key for evolution of new therapies. It would be possible to address key targets to prevent organ damage, thereby inhibiting progression toward liver failure, which will ultimately reduce the requirement for liver transplantation. We have recently shown that reduction in intrahepatic dendritic cells and increased interferon gamma (IFN-γ)-producing CD8+ T cells has an important role in hepatocellular injury which correlates with high mortality in ACLF (3).

Neutrophils are among first cell of defense recruited at the site of infection where they eliminate pathogen by phagocytosis (4), produce large amount of reactive oxygen species (ROS) (5), and contribute in healing and removal of cell debris. Though, high ROS will induce inflammation, tissue damage (6), and ultimately organ failure (7), in acute liver failure (ALF), neutrophils are served as prognostic marker of disease severity and outcome (8). Studies have shown neutrophil dysfunction with high spontaneous oxidative burst and reduced phagocytic activity in patients with cirrhosis (9) and alcoholic hepatitis (10), which was in turn associated with greater risk of infection and mortality. A recent study demonstrated an intravascular gradient of chemokines and mitochondria-derived formyl peptides direct neutrophils to sites of liver necrosis by CXCR2 and formyl peptide receptor 1, which induces injury and systemic inflammation during ALF in mice (11).

Chemokine receptors CXCR1 and CXCR2 are G-protein coupled receptors which are predominantly expressed on neutrophils (12, 13). CXCR1 and CXCR2 drives neutrophil migration in response to its ligand CXCL8/IL-8 (14), produced by many of the hepatic cells, including hepatocytes, stellate cells, and endothelial cells along with Kupffer cells, and act as an important component of hepatic inflammatory response during ischemia/reperfusion (15).

SCH 527123 is a novel allosteric antagonist of both CXCR1 and CXCR2 which inhibit chemokine binding to (and activation of) these receptors in an insurmountable manner and inhibit signal transduction, neutrophil chemotaxis, and myeloperoxidase release. It has been reported that blockade of CXCR1 and CXCR2 using SCH 527123 antagonist potentially inhibits human colon cancer liver metastasis (16) and also sensitizes cells to oxaliplatin (17). Moreover, CXCR1 and CXCR2 antagonist are also used to treat human melanoma (18), lung (19), breast (20), and pancreatic cancer (21) growth by inhibiting tumor cell proliferation and suppression of angiogenesis and metastasis.

Despite the fact that neutrophils are potentially involved in the pathogenesis of various liver diseases, their role in ACLF remains poorly understood. In the present study, comprehensive neutrophil functions were investigated in hepatitis B virus (HBV)-related ACLF and compared with chronic hepatitis B (CHB) and healthy controls (HC). Further to rule out any etiological-based variation in ACLF, alcohol-related ACLF patients were also included. Here, we identify a crucial role of neutrophils and CXCR1/2 signaling in propagating hepatic injury and further disease progression in ACLF patients, which was markedly abrogated after CXCR1/2 blockade with SCH 527123 antagonist. Therefore, the results of this study suggest CXCR1/2 as a potential therapeutic target for ACLF patients.

## Materials and Methods

### Patients

This was a cross-sectional study performed at a single tertiary care center. A total of 58 ACLF patients admitted to the Department of Hepatology, Institute of Liver and Biliary Sciences, were consecutively screened. Thirty-six ACLF patients (17 HBV related and 19 alcohol related) fulfilling ACLF diagnostic criteria were enrolled, and 22 patients were excluded as per the exclusion criteria. As all ACLF patients belong to the Asian region, Asian Pacific Association for the Study of the Liver criteria was considered for characterization (22, 23). In brief, the criteria was: a patient with an acute hepatic insult manifested as jaundice (serum bilirubin ≥5 mg/dl, 85 µmol/l) and coagulopathy [International normalized ratio (INR) ≥1.5 or prothrombin activity <40%] complicated within 4 weeks by ascites and/or encephalopathy (as determined by clinical examination), with previously diagnosed or undiagnosed chronic liver disease/cirrhosis. All ACLF (both HBV and alcohol related) and CHB patients underwent liver biopsy. ACLF patients had coagulopathy and ascites, thus transjugular liver biopsy (TJLB) was performed in these patients; however, in CHB, percutaneous liver biopsy was done. TJLB confirmed that all ACLF patients had cirrhosis. The diagnosis of cirrhosis was based on clinical, endoscopic, histopathological, and radiological criteria.

#### Hepatitis B Virus-Related ACLF (HBV-ACLF: *n* = 17)

Serological profile showed all HBV-ACLF patients were HBs antigen positive with raised HBV DNA >2 × 10^4^ IU/ml.

#### Alcohol-Related ACLF (Alcoholic-ACLF: *n* = 19)

Alcohol-related ACLF patients were having alcoholic hepatitis which was confirmed by histological evidence. These patients had a history of continued alcohol intake >2 years, with last intake within 1 month and were negative for other etiologies. During study, all patients were abstinent from alcohol.

#### Exclusion Criteria

ACLF patients coinfected with other hepatotropic viruses, including hepatitis A, C, D, E, or human immunodeficiency virus, and suspected autoimmune hepatitis (antinuclear antibody/ASMA-positive in titers ≥1:80 and/or IgG ≥1.5 times upper limit of normal) were excluded. Patients having any other illness within at least preceding 12 months, coexistent hepatocellular carcinoma, portal vein thrombosis, associated cardiovascular comorbidities, grade 3 or 4 hepatic encephalopathy, history of blood transfusion, recent variceal bleed, other cause of chronic liver failure, any concurrent evidence of sepsis confirmed by culture positivity, or presence of spontaneous bacterial peritonitis or any other microbial infection, intended for liver transplantation, uncontrolled hypertension, and those who refused to participate were excluded. Samples were collected from all the patients before any treatment, just to avoid drug-mediated alterations in neutrophil phenotype or function.

#### Chronic Hepatitis B (*n* = 42)

Chronic hepatitis B patients with HBs Ag positive and raised ALT (>1.2 × U/l) for at least 6 months and having HBV DNA >2 × 10^4^ IU/ml were taken. Liver biopsy confirmed that none of the patient had cirrhosis.

#### Healthy Control (*n* = 18)

Age- and sex-matched non-alcoholic healthy individuals with no past and present history of liver disease were taken.

### Blood and Liver Tissue Samples

Peripheral blood (20–30 ml) was collected in EDTA tubes. However, for phagotest and oxidative burst test, 2 ml blood was collected separately in sodium heparin tube, as per the requirements. Liver biopsy was collected in RPMI 1640 medium as well as RNA later for flow cytometry and reverse transcriptase polymerase chain reaction (RT-PCR), respectively. For immunohistochemistry (IHC), liver biopsy was collected in formalin.

### Isolation of Neutrophil Granulocytes

Neutrophils were isolated from ACLF (HBV and alcohol related), CHB, and HC groups by density gradient centrifugation using granulocyte separation media (Granulosep™ GSM 1119) and lymphocyte separation media (Hisep™ LSM 1077) (Himedia USA). In brief; 10 ml of GSM was transferred to a 50-ml tube and overlaid with 10 ml of LSM, then PBS -diluted blood was transferred, and tubes were centrifuged at 700 × *g* for 30 min. After centrifugation, two distinct layers were obtained. Neutrophils were taken out from lower layer leaving lymphocytes/mononuclear cells in upper layer and washed with PBS. To check the purity of isolated neutrophils, CD11b and CD16 (eBioscience, San Diego, CA, USA) staining was done and analyzed by flow cytometry. The purity of neutrophils exceeded 90%. The viability of the neutrophils was determined by LIVE/DEAD Fixable Blue Stain (ThermoFisher Scientific, MA, USA) and/or trypan blue exclusion assay and found to be 96%.

### Isolation of Liver-Infiltrating Leukocytes (LILs)

Liver-infiltrating leukocytes were isolated by mechanical disruption of liver biopsy in Medimachine for 1 min (BD Biosciences, San Jose, CA, USA) according to the technical instructions of the manufacturer. Cells were collected in complete RPMI 1640 media (10% fetal bovine serum, 2 gm/l sodium bicarbonate, and 1% penicillin/streptomycin) (Himedia, USA), and further experiments were performed.

### Flow Cytometry

Peripheral blood and LILs were incubated with anti-human CD11bPECY-7, CD16V450 (eBioscience, San Diego, CA, USA), CXCR1APC, CXCR2PE antibodies (BD Biosciences, San Diego, CA, USA), and LIVE/DEAD Fixable Blue Stain (ThermoFisher Scientific, MA, USA) in a 96-well plate for 20 min at 4°. CD14 Alexa Fluor (eBioscience, San Diego, CA, USA), CD56 BV650 (Biolegend, CA, USA), and CD68 fluorescein isothiocyanate (FITC) (Bio Rad, CA, USA) antibodies were also added to exclude monocytes, natural killer (NK) cells, and macrophages during analysis, as these cells also express CD11b and/or CD16. Red blood cells (RBCs) were lysed for 15 min using 1× lysing solution (BD Biosciences, San Diego, CA, USA). Peripheral blood cells and LILs were then washed twice with 1× PBS and resuspended in 1× PBS with 0.1% paraformaldehyde for acquisition on BD LSR II flow cytometer (BD Biosciences, USA). Data analysis was done using Flowjo version 8.8.7 software.

### Immunohistochemistry

Immunohistochemistry was performed on formalin fixed paraffin embedded liver tissue sections (2.5 µm thick) by using standard pathology laboratory protocols. Sections were deparaffinized in xylene and rehydrated through graded alcohol (100, 90, 80, and 70%) to PBS. Endogenous peroxidase was blocked by incubating the tissue sections in 15% hydrogen peroxide (H_2_O_2_) for 10 min and then kept in boiling sodium citrate buffer (pH 6.0) for 10 min for antigen retrieval, cooled in running water, and two washes in Tris buffer saline (TBS, pH 7.6). One drop of protein fixer was placed on tissue section for 10 min and then drained. Sections were incubated overnight with primary anti-human antibodies for CXCR1, CXCR2 (eBioscience USA, 1:50 dilution) (GeneTex USA, 1:50 dilution), caspase-3 (GeneTex; USA, 1:50 dilution) and receptor-interacting protein kinase 3 (RIP-3) at 4°, washed twice with TBS, and 30 µl of polymer super enhancer (secondary antibody) (SuperSensitive™ Polymer-HRP IHC Detection System/DAB large volume Biogenex, USA) was applied for 20–30 min at room temperature in a humid chamber. After washing twice with TBS, 30 µl of horseradish peroxidase, a tertiary antibody, was applied, which detects a small amount of a specific protein by producing a detectable signal. Then, 30 µl of diaminobenzidine was applied as a chromogen for 5–10 min and then washed with running water. Sections were counter stained with Mayer’s hematoxylin for 1 min, washed, and mounted in DePex resinous mounting medium. Slides were then analyzed under microscope at 20×. Further, scoring of IHC data was done where a numerical value for overall intensity [intensity score (IS)] was given on a scale of 0, 1, 2, and 3 for none, light, medium, and dark, respectively and mean IS was calculated.

### RNA Isolation

Total RNA was isolated from liver biopsy using AmbionmirVana™miRNA isolation kit (Ambion, USA). In brief, liver biopsies were lysed using 300 µl of lysis buffer and vortexed for 30 s; microRNA homogenate additive was added and incubated for 10 min. Three hundred microliters of phenol–chloroform were added and centrifuged (5 min at 13,000 rpm). Upper layer was taken out, absolute alcohol was added, and samples were transferred to the filter cartridge containing tube and spun (30 s at 10,000 rpm). Flow through was discarded, and cells were washed with 500 and 700 µl of wash buffer 1 and 2, respectively. Total RNA was eluted out in 20 µl of elution buffer. RNA concentration was measured on a nanodrop 2000 spectrophotometer (Thermo Scientific, USA). RNA integrity and quality was checked by bioanalyser (Agilent 2100 Bioanalyzer) (Agilent technologies USA).

### cDNA Synthesis

cDNA was synthesized by reverse transcriptase polymerase chain reaction using the following method:1 μl of random hexamer (0.2 µg/µl) (Fermentas, USA) was added in 300 ng of total RNA. Samples were incubated in mastercycler gradient PCR machine (Eppendorf, USA) at 65°C for 5 min then 4° for infinity. Two microliters of deoxynucleotides mix (dNTPs, 10 mM) (Fermentas USA), 0.5 µl of RNase inhibitor (20 U/μl) (Fermentas USA), 1 µl of reverse transcriptase (200 U/μl) (Fermentas USA), and 1.5 µl nuclease-free water were used. Samples were processed in a PCR machine for 1 h at 42°C, followed by 10 min incubation at 70°C and then reactions were stopped at 4°C.

### Determination of Relative mRNA Expression by Quantitative Real-time RT-PCR

Relative mRNA expression of CXCR1, CXCR2, CXCL8/IL-8, caspase-3, and RIP-3 was determined in liver biopsy of HBV-ACLF, alcoholic-ACLF, and CHB patients. Reactions were performed in duplicate with a 5-µl reaction volume using 2.5 µl of fluorogenic dye SYBR Green (2×) as a double-strand DNA-specific binding dye (Applied Biosystems USA), 0.25 µl of forward and reverse primer each (10 pmol/μl) (Biolinkk India), 1 µl of cDNA, and 1 µl of nuclease-free water. RT-PCR was carried out on an Applied BioSystems (ABIviia 7) thermocycler (Applied Biosystems, Inc., USA) using following condition: 95°C for 10 min, followed by 40 cycles of 95°C for 15 s and 60°C for 1 min, followed by a hold at 4° C. 18S was used as endogenous control. The absence of non-specific primer-dimer products was verified by melting curve analysis. Results were expressed in terms of relative mRNA expression normalized to 18S and calculated by using formula 2^−ΔCt^. Primer sequences have been listed in Table 1.

**Table 1:** **List of primers used in the study**.

S. No.	Primer name	Forward sequence	Reverse sequence
1	CXCR1	*TTTGTTTGTCTTGGCTGCTG*	*AGTGTACGCAGGGTGAATCC*
2	CXCR2	*ACAGCTACTTGGGAGGCTGA*	*TGCAGTGGTCACACCATTTT*
3	CXCL8/IL-8	*TAGCAAAATTGAGGCCAAGG*	*AAACCAAGGCACAGTGGAAC*
4	Caspase-3	*TTCGTGAGTGCTCGCAGCTCA*	*CCACCGAAAACCAGAGCGCC*
5	Receptor-interacting protein kinase 3	*CTTCCAGGAATGCCTACCAA*	*TCCATTTCTGTCCCTCCTTG*
6	18S	AAGTACGCACGGCCGGTACA	AGCGCCCGTCGGCATGTATT

### Oxidative Burst Test

Neutrophil’s ROS production was quantified by the burst test kit (Orpegen Pharma Heidelberg Germany). In brief, 100 µl of heparinized peripheral whole blood was incubated with 20 µl (2 × 10^7^ cells) of either unlabeled opsonized *E. coli* as particulate stimuli or wash buffer as a control. Blood samples were treated with 20 µl (0.32 mM) of either phorbol 12-myristate 13-acetate (PMA: 200× stock solution, 1.62 mM, 5 µl of stock solution was diluted in 1 ml of wash buffer and then 20 µl was used) or 20 µl (0.2 mM) of chemotactic synthetic peptide *N*-formyl-MetLuePhe (fMLP: 200× stock solution, 1 mM) to assess high and low oxidative burst capacity, respectively. Cells were incubated for 20 min at 37° in water bath, 20 µl of substrate (dihydrohodamine-123) was then added, and samples were further incubated for 20 min. RBCs were lysed, and after two washes with wash buffer, cells were stained for neutrophil markers mentioned above. After washing with PBS, cells were analyzed by flow cytometry. Oxidative burst was quantified according to the percentage of CD11b+ CD16+ neutrophils producing ROS. The production of ROS was measured by flow cytometry using the oxidation of dihydrohodamine-123 to rhodamine which emits green fluorescence.

### Phagotest to Measure Neutrophil Phagocytic Activity (NPA)

Peripheral blood was collected in sodium heparin tubes and cooled on ice for 10 min. Phagocytosis was quantified using the Phagotest kit (Orpegen Pharma Heidelberg Germany), which uses FITC-labeled opsonized *E. coli* bacteria. In brief, 20 µl of FITC-labeled opsonized *E. coli* (2 × 10^7^) was mixed with 100 µl of whole blood sample and further incubated in a water bath at 37°C for 20 min. Hundred microliters of ice-cold trypan blue solution were used to quench the fluorescence of bacteria bound to the cell surface. Cells were washed twice at 270 × *g* for 5 min with wash buffer provided in the kit; RBC were lysed using lysing solution. After two washes, cells were stained for neutrophil markers using CD11b PECY-7 and CD16 PE antibodies and acquired on a flow cytometer. Granulocytes were gated according to forward and side scatter, and then neutrophils were gated on the basis of CD11b and CD16. NPA was detected by measuring the fluorescence of FITC bound to opsonize *E. coli* by flow cytometry. NPA was expressed as the percentage of neutrophils undergoing phagocytosis.

### Immunofluorescence Microscopy

Neutrophil Phagocytic Activity was also detected by immunofluorescence microscopy for which neutrophils were incubated with 20 µl of FITC-labeled opsonized *E. coli* (2 × 10^7^) for 20 min and then analyzed by inverted microscope at 20× (Nikon Inverted Microscope eclipse Ti-S, software NIS-Elements BR 3.1, Nikon Instruments Inc., USA) under blue filter [excitation filter 455/70 (420–490) nm, dichromatic mirror (505 nm) barrier filter (520 nm)]. Fluorescence intensity of cells was calculated by ImageJ software, and mean fluorescence intensity score is displayed.

### Cytokines/Chemokines Analysis

Cytokines/chemokines were evaluated in HC, CHB, and ACLF groups. Cell isolated from peripheral blood were stimulated for 30 min, 1, 3, 6, 12, and 18 h and cytokine/chemokines production was measured. The cytokine/chemokine production was started at 3 h and reached maximum at 18 h. Therefore, all the further experiments were performed at 18 h. Cells were stimulated with LPS (100 ng/ml) for 18 h. Brefeldin A at a concentration of 2 µg/ml (Sigma-Aldrich USA) was added after 2 h incubation with LPS. Plates were then centrifuged for 7 min at 1,300 rpm. Cells were surface stained with CD11b and CD16 antibodies, washed, permiablized with cytofix/cytoperm and washed twice with 100 µl Perm/Wash buffer (BD Biosciences, USA). Intracellular antibodies CXCL8/IL-8, IL-6, IL-17, IL-23, CCL-20 (Biolegend, CA, USA), and GM-CSF (eBioscience, USA) were added and incubated for 20 min. After two washes, cells were resuspended in 0.1% paraformaldehyde in PBS prior to analysis.

### Neutrophils, HepG2, and HepG2.2.15 Cells Coculture Assay

To determine the mechanisms of neutrophil-mediated liver injury, *in vitro* coculture assay was performed. HepG2 and HepG2.2.15 cells were maintained in a humidified 5% CO_2_ atmosphere at 37°C in complete DMEM medium (10% fetal bovine serum, 1% penicillin/streptomycin) (Himedia, USA) HepG2 cells (1 × 10^5^) were cultured with neutrophils isolated from HC, CHB, and ACLF patients at a ratio of 10:1, 2:1, and 1:1 for 18 h and stained with CD11b PECY-7 antibody (eBioscience, San Diego, CA, USA), followed by staining with annexin V APC (apoptosis) (BD Pharmingen™, San Diego, CA, USA) and propidium iodide (PI: necrosis) (BD Pharmingen™, San Diego, CA, USA). In another set of experiment, for imitating the *in vivo* environment of HBV replication, HepG2.2.15 cells (1 × 10^5^) were cultured with HC, CHB, and ACLF neutrophils in above mentioned ratio.

### Culture of HepG2 and HepG2.2.15 Cells with Neutrophil’s Supernatant

To demonstrate whether neutrophils induce cell death only through contact-dependent mechanisms or the neutrophil’s inflammatory mediators are also conductive toward cell death, culture of HepG2 and HepG2.2.15 cells was done in the presence and absence of ACLF neutrophil’s condition medium. For this, 1 × 10^5^ HepG2 and HepG2.2.15 cells were cultured with the supernatant collected from ACLF neutrophils with and without LPS stimulation (100 ng/ml) for 18 h. HepG2/HepG2.2.15 cells cultured in the presence of LPS were taken as control. Subsequently cells were stained with annexin V APC and PI, acquired on flow cytometer (BD FACS Calibur™, San Diego, CA, USA). Apoptosis and necrosis of HepG2 and HepG2.2.15 cells was analyzed with flowjo software according to annexin V- and PI-positive cells, respectively.

### CXCR1 and CXCR2 Blockade Assay

To prove that CXCR1 and CXCR2 induce cell death through contact-dependent and -independent mechanisms, CXCR1 and CXCR2 blockade assay was performed. For this experiment, 18-h coculture of HepG2 and HepG2.2.15 with neutrophils from HC, CHB, and ACLF (1:1 ratio) was done in the presence or absence of CXCR1 and CXCR2 antagonist (SCH 527123, IC50: CXCR1—42 nm, CXCR2—3 nm) (Apexbio Bostan, MA, USA) at a concentration of 100 nM. SCH 527123 stock was prepared in dimethyl sulfoxide, and further working stock was made in 1× PBS with 0.1% bovine serum albumin. To demonstrate whether CXCR1 and CXCR2 had any effect on the production of inflammatory mediators, neutrophils from HC, CHB, and ACLF groups were incubated with and without CXCR1 and CXCR2 antagonist SCH 527123 (100 nm) along with *E. coli* stimulation (100 ng/ml) for 18 h. Brefeldin A (2 µg/ml) was added after 2 h incubation. After the completion of the incubation time, cells were washed with 1× PBS and surface stained with CD11b and CD16 antibodies. After a PBS wash, cells were fixed/permiablized, and intracellular staining was performed with CXCL8/IL-8, IL-6, IL-17, IL-23, CCL-20, and GM-CSF antibodies. Cells were then acquired on flow cytometer and analyzed by flowjo software. The effect of CXCR1 and CXCR2 blockade on ROS generation was also detected in all groups after *E. coli* stimulation. Further to find out the time point, when the decrease in ROS started, ACLF neutrophils were stimulated with *E. coli* in the presence and absence of CXCR1 and CXCR2 antagonist for 0, 3, 6, and 18 h. Cells were then incubated with rhodamine for 20 min and washed twice with wash buffer and resuspended in 1× PBS. ROS was measured by immunofluorescence microscopy (Nikon Inverted Microscope eclipse Ti-S, software NIS-Elements BR 3.1) under blue filter [excitation filter 455/70 (420–490) nm, dichromatic mirror (505 nm) barrier filter (520 nm)]. Fluorescence intensity of cells was calculated by ImageJ software, and mean fluorescence intensity (MFI) is displayed.

### Statistical Analysis

Data were analyzed using Graph Pad Prism 5 and SPSS software. Values are displayed as median and interquartile range or mean and SD. Statistical differences were assessed by performing one-way analysis of variance (Bonferroni’s post test), and Kruskal–Wallis with Dunn’s multiple comparison test for comparing three or more groups. Comparison between two groups was done by Mann–Whitney and Student’s *t*-test.

Multivariate analysis was performed to study the role of different neutrophil factors in predicting mortality in ACLF patients. Receiver operating characteristics (ROC) curve was used to determine the cutoff point for absolute neutrophil count (ANC) and CXCL8/IL-8in predicting mortality. Any relationship between neutrophil phenotypic and functional parameters with clinical disease severity was analyzed using Pearson correlation coefficient. Values of *p* < 0.05 were considered significant.

## Results

### Baseline Demographic and Clinical Parameters of the Study Subjects

Baseline characteristics of the study population have been listed in Table 2. Alanine aminotransferase (ALT) was higher in ACLF groups than HC, however, comparable between ACLF and CHB. Aspartate transaminase (AST), serum bilirubin, and creatinine level were higher in ACLF groups than CHB and HC. Albumin level was lower in ACLF groups. Total leukocytes count, ANC, and percentage of ANC (percent ANC represents the percentage of neutrophils from total leukocyte count) were significantly higher in ACLF groups as compared to CHB and HC. No significant differences in biochemical parameters as well as International Normalized Ratio (INR), Child Turcotte Pugh (CTP), and Model of End Stage Liver Disease (MELD) scores were seen in ACLF patients with both etiologies.

**Table 2:** **Baseline characteristics of the study subjects**.

Parameters	Healthy controls (*n* = 26) (A)	Chronic hepatitis B (*n* = 42) (B)	HBV-ACLF (*n* = 17) (C)	Alcoholic-ACLF (*n* = 19) (D)	*p*-Value
Age (years)	35 (28–46)	36 (23–47)	42 (24–59)	42 (32–62)	0.23
Male:female	2:1	3:1	2:1	1:0	0.09
ALT (U/l)	19 (17–35)	62 (65–275)	69 (73–197)	72 (64–134)	0.04
AST (U/l)	23 (19–39)	75 (63–212)	109 (60–331)	93 (56–181)	0.002
S bilirubin (mg/dl)	0.6 (0.3–1.2)	1 (1–9)	17 (6–47)	18 (8–39)	0.0001
Albumin (g/dl)	4.3 (3.6–5)	4 (2–4)	2.5 (1–3)	2.6 (2–3)	0.02
Creatinine (mg/dl)	0.52 (0.42–0.77)	0.64 (0.48–0.91)	1 (0.71–1.23)	1 (0.7–1.13)	0.1
Platelets (×10^3^/mm^3^)	204 (155–376)	150 (45–233)	115 (90–170)	120 (40–213)	0.30
TLC (×10^3^/mm^3^)	5.5 (4.5–8.9)	6 (3.6–8.5)	7.3 (5–12)	10.5 (2–19)	0.02
ANC (×10^3^/mm^3^)	2.92 (2.2–4.4)	3.48 (2.1–4.6)	6.1 (2.4–9.4)	7.4 (1.3–14.7)	0.01
ANC (%)	52.45 (46–55)	58.5 (53–70)	72 (61–76)	74.5 (65–80)	0.006
Ascites (*n*)	–	None	17 (100%)	19 (100%)	–
CTP score	–	–	11 (9–12)	12 (11–13)	–
INR	–	–	2.12 (1.8–3.2)	2.4 (1.7–3.8)	–
MELD score	–	–	23 (21–33)	27 (24–34)	

*Values are expressed as median (range) and number (%)*.

*ALT, S bilirubin; TLC, total leukocyte count; ANC, absolute neutrophil count; CTP, Child Turcotte Pugh; INR, international normalized ratio; MELD, model of end-stage liver disease*.

*One-way analysis of variance test was performed to compare all the groups. *p* < 0.05 was considered statistically significant. No significant difference in any parameter was observed in hepatitis B virus (HBV)-ACLF and alcoholic-ACLF patients*.

### Neutrophils Were Increased in ACLF and Had High CXCR1 and CXCR2 Expression

**Figure 1:**
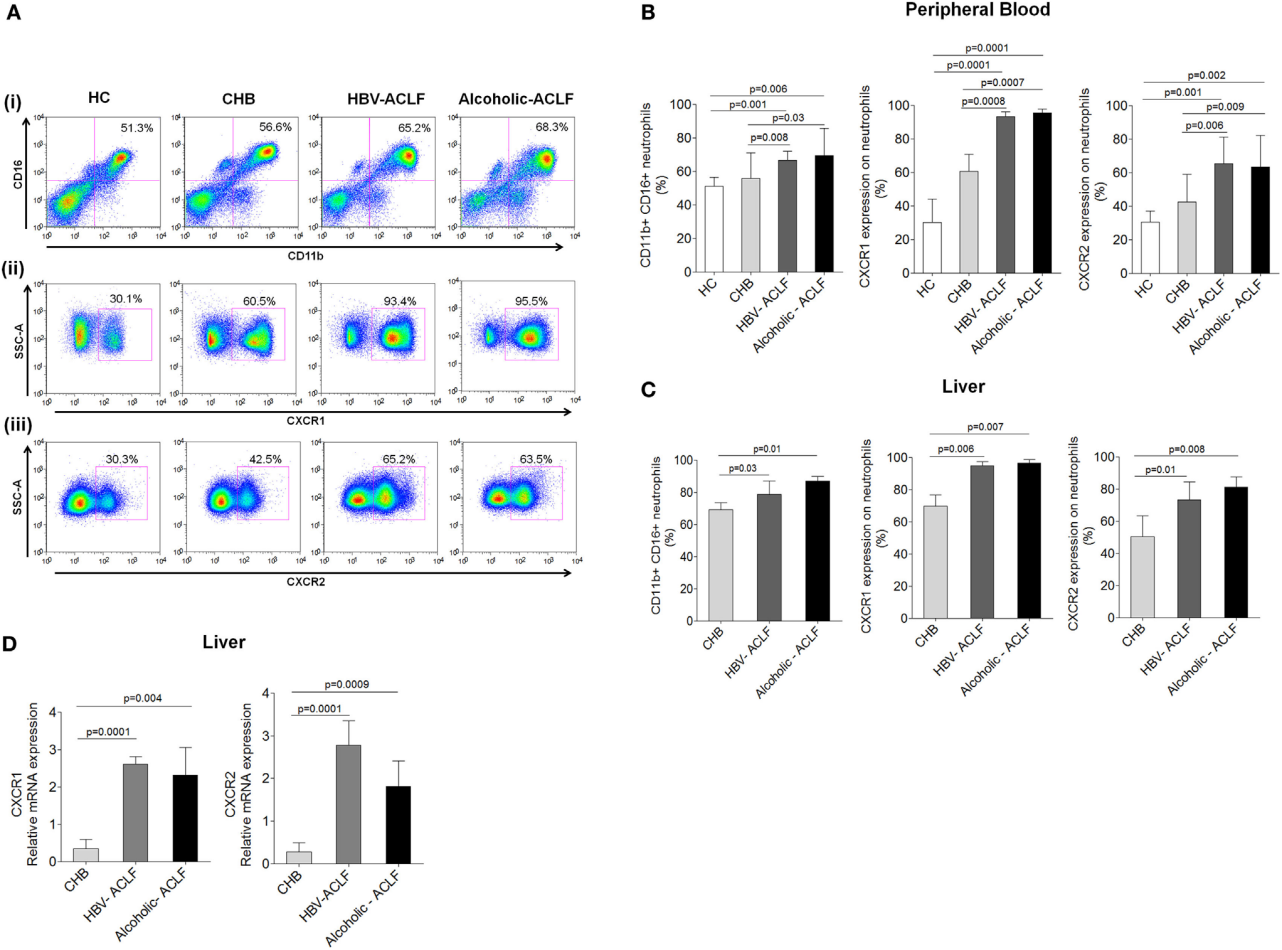
**Acute-on-chronic liver failure (ACLF) patients have increased neutrophils, which express high CXCR1 and CXCR2**. **(A)** Representative pseudo color plot indicate the percentage of (i) CD11b and CD16 double positive neutrophils, (ii) expression of CXCR1, and (iii) CXCR2 in peripheral blood. **(B,C)** Bar graphs demonstrate percentage of neutrophils, CXCR1, and CXCR2 in peripheral blood as well as liver. **(D)** RT-PCR results indicate intrahepatic relative mRNA expression of CXCR1 and CXCR2. Data are expressed as mean ± SD.

Neutrophil profile was examined in peripheral blood and liver tissue. Neutrophil gating strategy has been demonstrated Figure S1 in Supplementary Material. To specifically characterize neutrophils on the basis of CD11b and CD16 double-positive population, monocytes, NK cells, and macrophages which have some expression of CD11b and/or CD16 were excluded during analysis based on CD14, CD56, and CD68 positivity. Expression of CXCR1 and CXCR2 was analyzed on CD11b and CD16 double-positive neutrophils. Representative pseudo color plot indicate the percentage of (i) neutrophils (ii) expression of CXCR1, and (iii) CXCR2 in HC, CHB, HBV-ACLF, and alcoholic-ACLF patients (Figure 1A). In comparison to CHB and HC, neutrophil percentage and expression of CXCR and CXCR2 was significantly higher in peripheral blood of ACLF patients (Figure 1B). Further, ACLF patients also had increased neutrophils and CXCR1 and CXCR2 receptor expression in liver (Figure 1C). No significant differences were observed between both ACLF groups.

Expression of CXCR1 and CXCR2 was also analyzed in liver tissue by IHC. ACLF patients showed higher CXCR1 and CXCR2 expression in liver tissue than CHB. In ACLF groups, robust CXCR1 and CXCR2 expression was observed in necrotic areas of liver tissue (Figure S2A in Supplementary Material), which may suggest crucial involvement of CXCR1 and CXCR2 in tissue damage. Further, scoring of IHC data was done and mean IS was calculated. Out of 20 liver biopsies from ACLF (10 from each group), 18 scored positive and the mean IS of CXCR1 and CXCR2 expression was higher in ACLF groups than CHB (Figure 2B in Supplementary Material).

Relative mRNA expression of CXCR1 and CXCR2 was evaluated in the liver tissue of all patient groups by RT-PCR. In agreement with the flow cytometry and IHC data, CXCR1 and CXCR2 mRNA expression was significantly elevated in ACLF patients than CHB (Figure 1D).

### Increased Neutrophil Count Is Associated with Disease Severity and Mortality in ACLF

**Figure 2:**
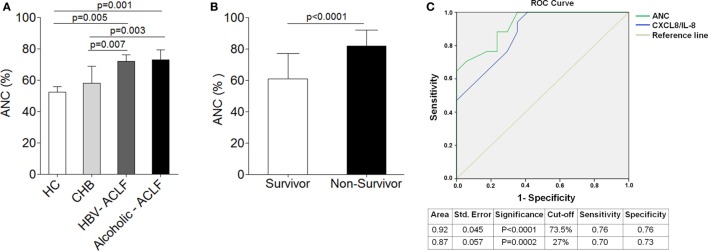
**Higher neutrophil counts are associated with disease outcome and mortality in acute-on-chronic liver failure (ACLF)**. **(A)** Absolute neutrophil count (ANC) was significantly high in hepatitis B virus (HBV)-ACLF (*n* = 17) and alcoholic-ACLF (*n* = 19) than chronic hepatitis B (CHB) (*n* = 42) and healthy controls (HC) (24). **(B)** Bar graph indicates ANC in ACLF survivors (*n* = 17) and non-survivors (*n* = 17). ANC was significantly higher in non-survivors. **(C)** Receiver operating characteristic curve of ANC and CXCL8/IL-8 in prediction of mortality. ANC had high area under the curve 0.92 with a sensitivity of 76.5% and specificity of 76.5 than CXCL8/IL-8.

To ensure flow cytometry results which indicate high percentage of neutrophils in both ACLF groups in comparison to CHB, we analyzed ANC in all groups. The data revealed significant increased ANC in ACLF patients than CHB and HC (Figure 2A). ANC was also analyzed in clinically severe ACLF patients. The results demonstrated that ACLF patients with high bilirubin [>20 mg/dl, median value 27 (22–47)] had significant increase (*p* < 0.03) in ANC as compared to those with low bilirubin [<20 mg/dl, median value 13 (6–18)]. Similarly, patients with high MELD score ≥30 (30–34) had higher ANC (*p* < 0.04) than those with low MELD score (21–29). ACLF patients with coagulation failure [INR >2.5 (2.7–3.8)] also showed increased ANC (*p* < 0.02) than with low INR value (1.7–2.5).

Absolute neutrophil count was also determined in ACLF survivors/non-survivors (*n* = 34). Most importantly, ACLF non-survivors (*n* = 17) had significant increased ANC (27.8%) as compared to survivors (*n* = 17) (Figure 2B). Further, to determine whether any alteration in neutrophils could predict mortality in ACLF, we performed multivariate analysis of different neutrophil factors, including ANC, CXCR1, CXCR2, phagocytic activity, ROS, CXCL8/IL-8, IL-6, IL-17, IL-23, CCL-20, and GM-CSF. Multivariate analysis revealed ANC [odd ratio = 1.25 (CI = 1.08–1.45), *p* = 0.003] as well as CXCL8/IL-8 [odd ratio = 1.19 (CI = 1.04–1.36), *p* = 0.009] as a predictor of mortality in ACLF. For the prediction of mortality, the sensitivity and specificity of ANC and CXCL8/IL-8 was investigated by ROC. We established the cutoff point of ANC > 73.5 (CI 95% 0.828–1.00) had a sensitivity and specificity of 76% in predicting mortality. However, CXCL8 cutoff point of >27 (CI 95% 0. 763–0.988) had a sensitivity of 70% and specificity of 73% in prediction of mortality. Therefore, ROC analysis confirmed that ANC had more sensitivity and specificity in prediction of mortality than CXCL8/IL-8 which suggests ANC as a better predictor of mortality (Figure 2C).

### Neutrophils Produce High Amount of ROS and Exhibit Reduced Phagocytic Activity in ACLF Patients

**Figure 3:**
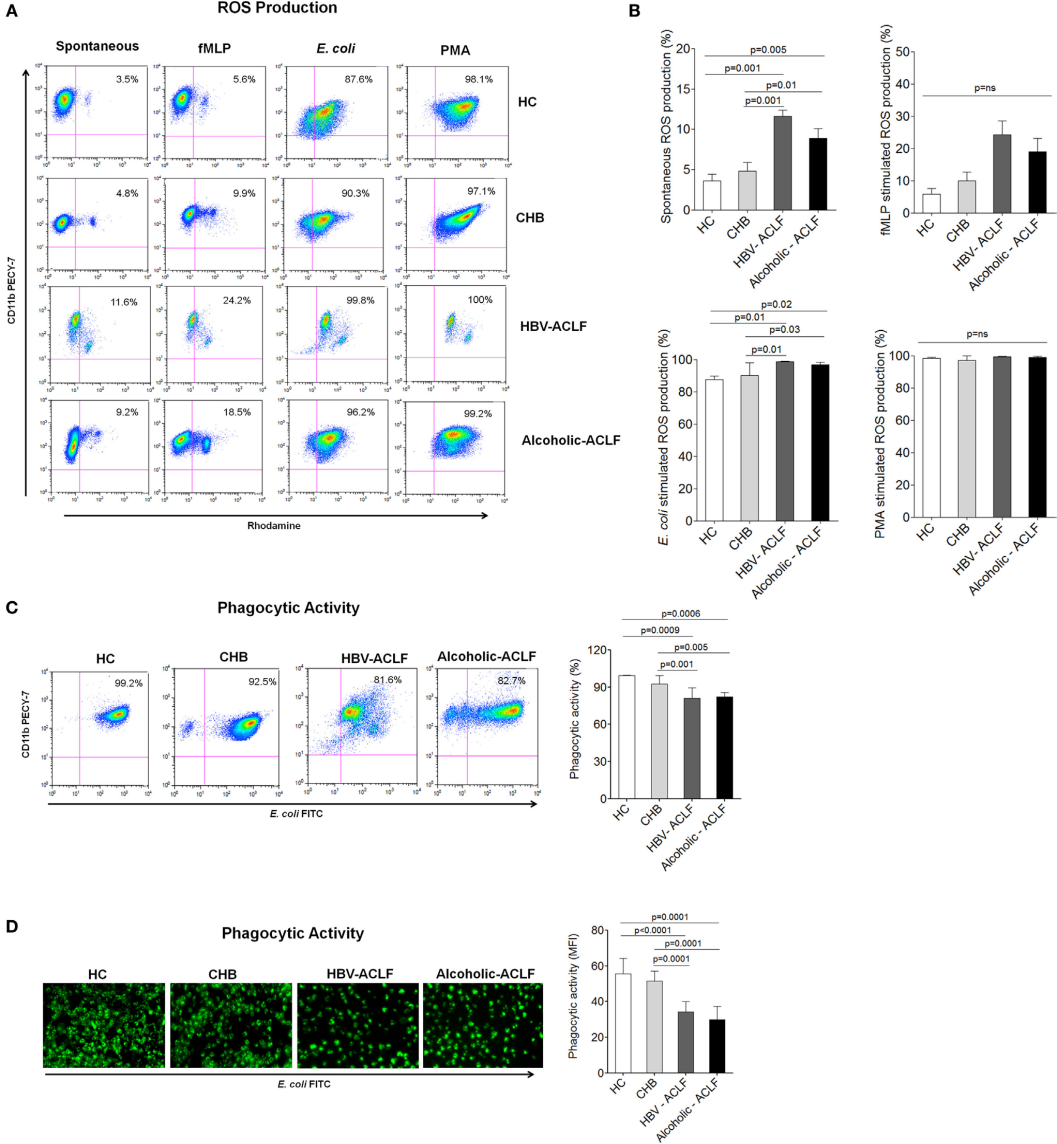
**Neutrophils from acute-on-chronic liver failure (ACLF) patients have high reactive oxygen species (ROS) production and reduced phagocytic activity**. **(A)** Flow cytometry plot represents spontaneous as well as fMLP-, *E. coli-*, and PMA-stimulated ROS production in patient groups and healthy controls (HC). **(B)** Results showed highest spontaneous and *E. coli*-stimulated ROS production in ACLF [hepatitis B virus (HBV)-ACLF *n* = 17, alcoholic-ACLF *n* = 19] than chronic hepatitis B (CHB) (*n* = 30) and healthy controls (HC) (*n* = 18). **(C)** Dot plot and bar graph showed reduced neutrophils phagocytic activity (NPA) in ACLF groups. In representative dot plot, *x* and *y* axes indicate *E. coli* FITC and CD11bPECY-7, respectively. Values mentioned in the upper right quadrant indicate NPA. **(D)** Immunofluorescence also showed reduced phagocytic activity in ACLF (magnification: 20×). Mean fluorescence intensity of phagocytic activity was calculated by ImageJ software and found lower in ACLF patients.

Increased ROS may potentiate tissue damage; therefore, we measured ROS production by peripheral blood neutrophils. Flow cytometry results demonstrated high spontaneous and *E. coli*-stimulated ROS production in ACLF patients in comparison to CHB and HC. When low and high burst capacity of neutrophils was assessed following stimulation with fMLP and PMA, no remarkable difference in ROS production were observed between the different cohorts (Figures 3A,B).

In addition, we assessed NPA, an important function to remove infectious agents. Flow cytometry results showed strong reduction in NPA in ACLF patients as compared to CHB and HC (Figure 3C). NPA was also checked by analyzing the phagocytosis of FITC-labeled opsonized *E. coli* by immunofluorescence microscopy. The data showed reduced NPA in ACLF groups than CHB and HC. MFI of NPA was calculated by imageJ software and found to be significantly lower in ACLF groups (Figure 3D).

### Increased CXCL8 Along with Other Inflammatory Cytokines in ACLF Patients

**Figure 4:**
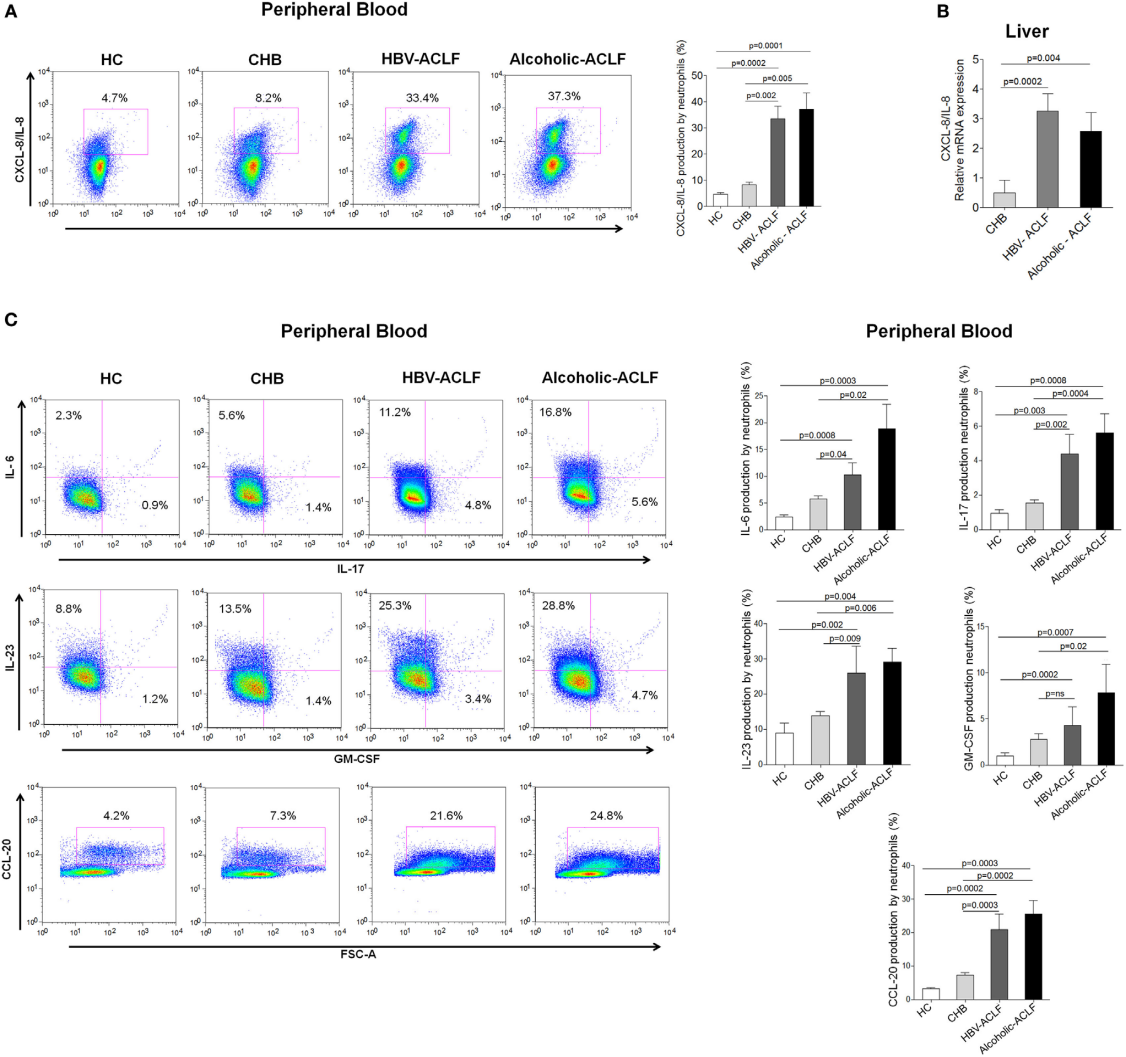
**High level of CXCL8/IL-8 (CXCR1/2 ligand) in acute-on-chronic liver failure (ACLF) patients**. **(A)** Flow cytometry results showed increased CXCL8/IL-8 production by peripheral neutrophils of ACLF patients compared to chronic hepatitis B (CHB) and healthy controls (HC). **(B)** Also, in liver, CXCL8/IL-8 relative mRNA expression was markedly upregulated in ACLF groups than CHB. **(C)** Flow cytometry plot and bar graphs illustrate increased production of other inflammatory cytokines/chemokines (IL-6, IL-17, IL-23, GM-CSF, and CCL-20) in peripheral blood of ACLF [hepatitis B virus (HBV)-ACLF *n* = 17, alcoholic-ACLF *n* = 19] compared to CHB (*n* = 30) and HC (*n* = 18).

CXCL8/IL-8 is a well-known CXCR1 and CXCR2 ligand, required for neutrophil activity and is associated with inflammation and tissue injury (25). We observed that peripheral blood neutrophils produce higher amount of CXCL8/IL-8 in ACLF patients than CHB and HC (Figure 4A). Importantly, intrahepatic CXCL8/IL-8 mRNA expression was significantly higher in ACLF groups than CHB (Figure 4B). Further, we evaluated the production of other inflammatory cytokines/chemokines in peripheral blood neutrophils which could be involved in liver injury. All tested inflammatory cytokines/chemokines, including IL-6, IL-17, IL-23, and CCL-20, were significantly increased in ACLF patients with both etiologies than CHB and HC. However, GM-CSF production was significantly elevated only in alcoholic-ACLF not in HBV-ACLF patients (Figure 4C).

### Neutrophil Functions Strongly Correlated with Clinical Parameters of Disease Severity

Importantly, CXCR1 expression significantly correlated with MELD and CTP score along with AST. CXCR2 also showed correlation with MELD score. Further, reduced NPA was significantly associated with MELD and CTP score though no correlation was seen with INR. Biochemical parameters, including ALT, AST, s bilirubin, and creatinine did not show any correlation with reduced phagocytic activity (Table 3). Importantly, CXCL8/IL-8 was positively associated with ALT, AST, and creatinine levels. Moreover, high CXCL8/IL-8 level was significantly correlated with the IL-6 (*p* < 0.009, *r*^2^ = 0.6). IL-23 level was strongly associated with CTP score (*p* < 0.04, *r*^2^ = 0.51) and INR (*p* < 0.03, *r*^2^ = 0.54). IL-23 also showed an association with IL-6 (*p* < 0.05, *r*^2^ = 0.5), CCL-20 (*p* < 0.02, *r*^2^ = 0.57), and IL-17 (*p* < 0.05, *r*^2^ = 0.5). In addition, IL-17 had a significant correlation with IL-6 (*p* < 0.006, *r*^2^ = 0.62) and CCL-20 level (*p* < 0.007, *r*^2^ = 0.6). Further, increased ROS was significantly associated with elevated level of G-MCSF (*p* < 0.05, *r*^2^ = 0.5).

**Table 3:** **Correlation of neutrophil functions with clinical disease severity indices**.

Neutrophils markers	ALT	AST	T Bil	Creatinine	International normalized ratio	Child–Turcotte–Pugh	Model of end stage liver disease
CXCR1	*r*^2^ = 0.21	*r*^2^ = 0.72	*r*^2^ = 0.13	*r*^2^ = 0.18	*r*^2^ = 0.32	*r*^2^ = 0.63	*r*^2^ = 0.45
	*p* = ns	*p* < 0.002	*p* = ns	*p* = ns	*p* = ns	*p* < 0.04	*p* < 0.05
CXCR2	*r*^2^ = 0.14	*r*^2^ = 0.22	*r*^2^ = 0.27	*r*^2^ = 0.31	*r*^2^ = 0.35	*r*^2^ = 0.09	*r*^2^ = 0.59
	*p* = ns	*p* = ns	*p* = ns	*p* = ns	*p* = ns	*p* = ns	*p* < 0.03
Neutrophil phagocytic activity	*r*^2^ = 0.29	*r*^2^ = 0.34	*r*^2^ = 0.23	*r*^2^ = 0.07	*r*^2^ = 0.11	*r*^2^ = 0.52	*r*^2^ = 0.5
	*p* = ns	*p* = ns	*p* = ns	*p* = ns	*p* = ns	*p* < 0.002	*p* < 0.04
CXCL-8	*r*^2^ = 0.51	*r*^2^ = 0.528	*r*^2^ = 0.17	*r*^2^ = 0.54	*r*^2^ = 0.07	*r*^2^ = 0.29	*r*^2^ = 0.33
	*p* < 0.03	*p* < 0.03	*p* = ns	*p* < 0.01	*p* = ns	*p* = ns	*p* = ns

### CXCR1/2 Receptors Blockade with SCH 527123 Antagonist Inhibits Contact-Dependent Cell Death

**Figure 5:**
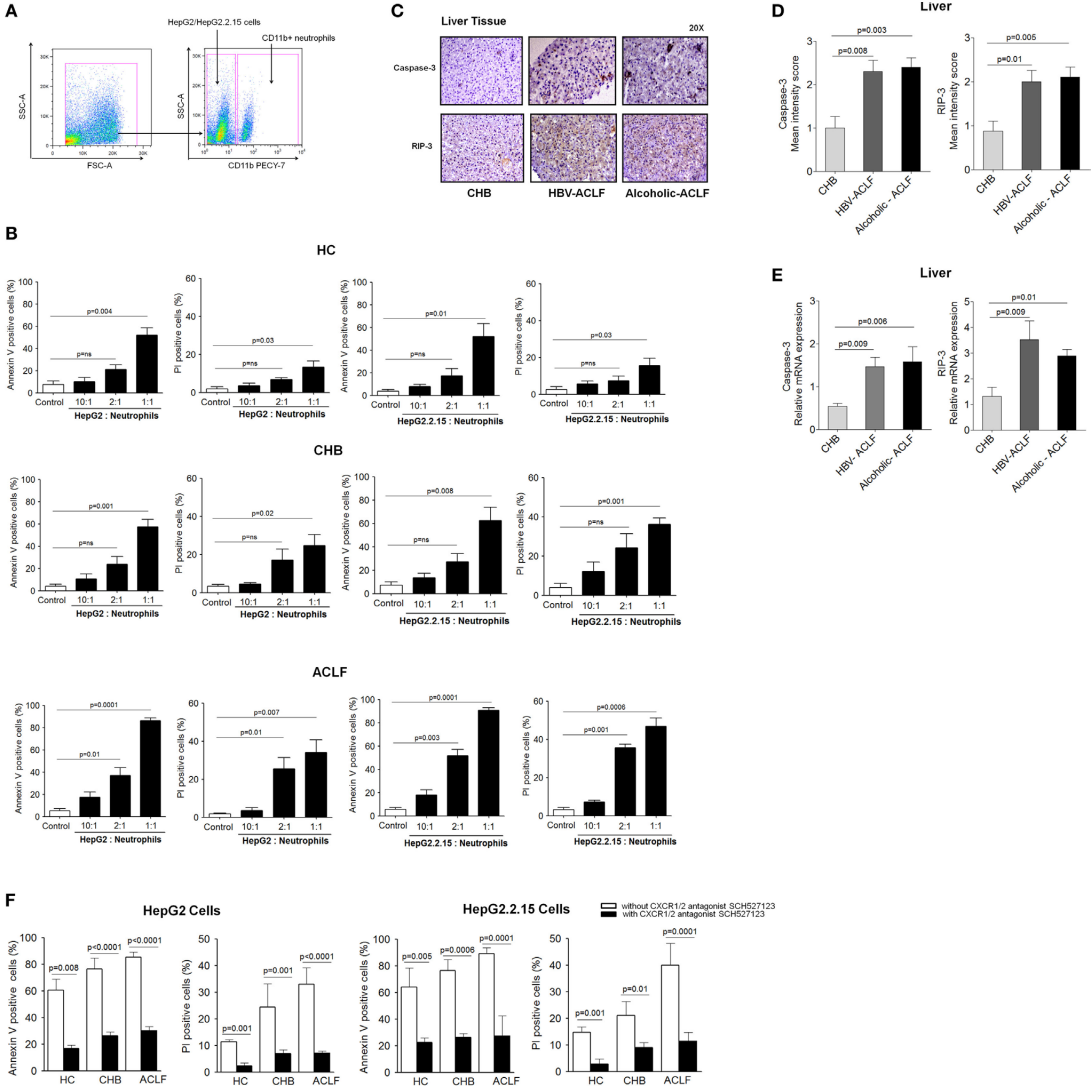
**CXCR1/2 receptors blockade with SCH 527123 antagonist prevent contact-dependent cell death**. **(A)** Gating strategy for neutrophil and HepG2/HepG2.2.15 coculture. For coculture experiments, neutrophils were isolated from health subjects and patient groups (*n* = 15). During quantification of cell death, neutrophils were excluded during flow cytometry analysis and only HepG2 and HepG2.2.15 cells were analyzed. **(B)** Coculture of HepG2 and HepG2.2.15 cells with increasing no. of neutrophils resulted in significant contact-dependent cell death of HepG2 and HepG2.2.15 cells through apoptosis and necrosis. **(C)** In liver tissue, hepatocytes showed intense caspase-3 and receptor-interacting protein kinase 3 (RIP-3) staining in acute-on-chronic liver failure (ACLF) (magnification: 20×). **(D)** In IHC staining, mean intensity score caspase-3 and RIP-3 was higher in ACLF groups than CHB. **(E)** RT-PCR data revealed high relative mRNA expression of caspase-3 and RIP-3 in ACLF. **(F)** CXCR1/2 blockade with SCH 527123 antagonist resulted in significant reduction of apoptotic (annexin V-positive cells) and necrotic (PI-positive cells) cell death.

Our data revealed high CXCR1/2 expression in ACLF patients. Further, IHC results demonstrated high expression of these receptors clustered to the necrotic areas of liver. To evaluate whether CXCR1/2 contribute to liver damage in contact-dependent mechanism, HepG2 and HepG2.2.15 cells (Hepatocyte cell lines) were cocultured with increasing numbers of CXCR1/2-expressing neutrophils, isolated from HC, CHB, and ACLF patients (HepG2: neutrophils ratios 10:1, 2:1, and 1:1). Viability of neutrophils was ensured at different time points to find out whether they would survive throughout the experiment or not. We observed that 90% of the neutrophils survived till 24 h (Figure 3 in Supplementary Material). Gating strategy of neutrophil and HePG2/HepG2.2.15 cells has been shown in Figure 5A. During flow cytometry analysis, neutrophils were excluded and only HepG2 and HepG2.2.15 cells were analyzed for quantification of cell death. Coculture of HepG2 and HepG2.2.15 cells with increasing number of CXCR1/2-expressing neutrophils resulted in significant cell death of both HepG2 and HepG2.2.15 cells through early apoptosis and necrosis. Notably even small number of neutrophils from ACLF patients were sufficient to induce cell death (Figure 5B). Further, to assure that high CXCR1/2 induces hepatocyte death; expression of caspase-3 (apoptosis) and RIP-3 (necrosis markers) was analyzed in the liver tissue of ACLF group and compared with CHB. IHC showed stronger caspase-3 and RIP-3 expression in ACLF patients than CHB (Figure 5C). Moreover, mean IS of caspase-3 and RIP-3 was higher in ACLF patients than CHB (Figure 5D). RT-PCR data also confirmed increased relative mRNA expression of caspase-3 and RIP-3 in the liver of ACLF patients as compared to CHB (Figure 5E).

Importantly, blockade of CXCR1/2 with SCH 527123 antagonist resulted in a significant reduction of apoptosis and necrosis of both HepG2 and HepG2.2.15 cells (Figure 5F), which confirms potential involvement of CXCR1/2 in mediating contact-dependent hepatic cell death. Therefore, the data suggest CXCR1/2 as a potential therapeutic target to control liver damage.

### Blockade of CXCR1/2 Receptors with SCH 527123 Antagonist Reduces the Production of Neutrophil’s Inflammatory Mediators and Prevents Contact-Independent Cell Death

**Figure 6:**
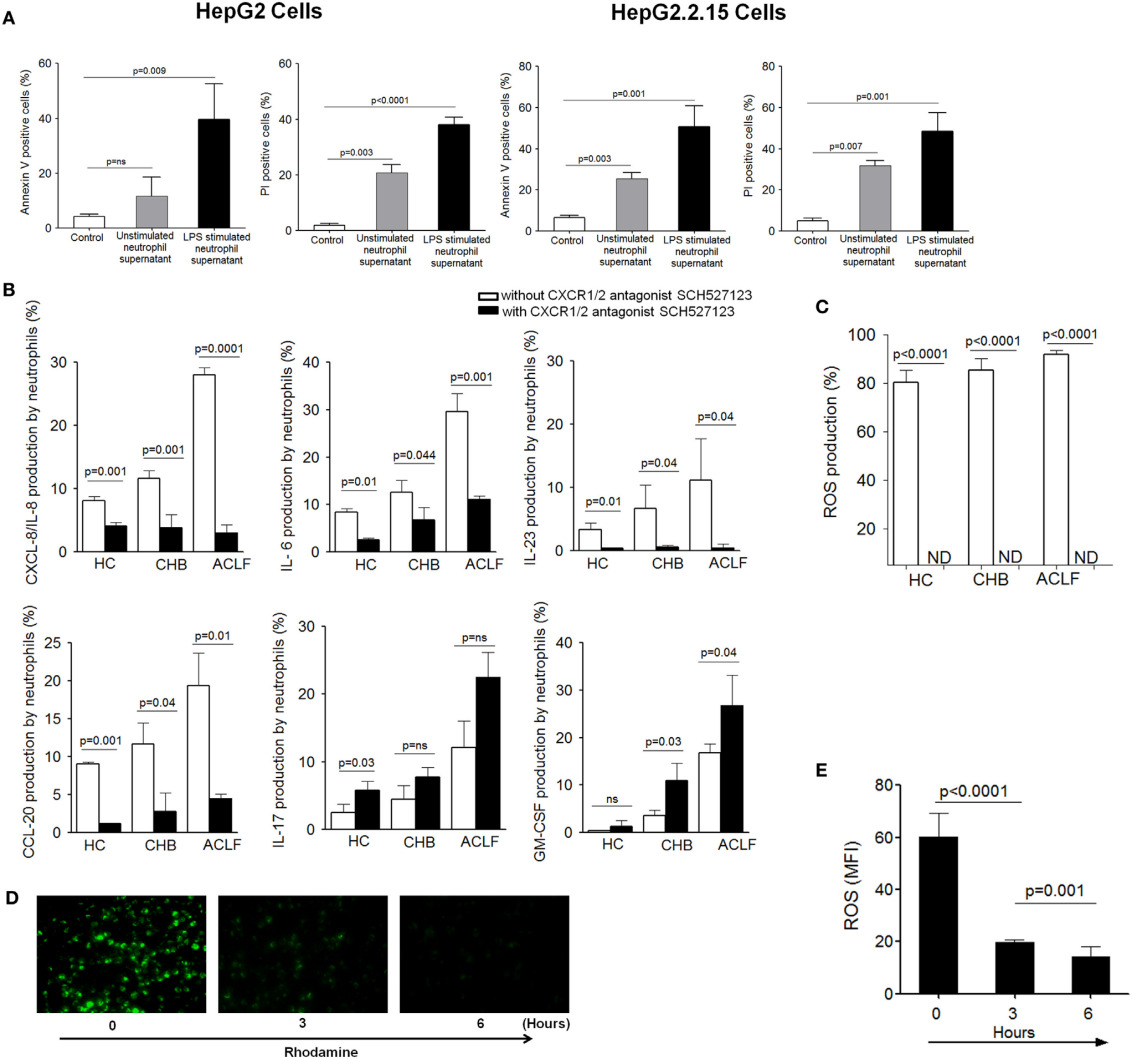
**Blocking of CXCR1/2 receptors inhibit production of neutrophil’s inflammatory mediators and reduce cell death**. **(A)** Culture of HepG2 and HepG2.2.15 cells with resting and LPS-activated neutrophil’s supernatant (from ACLF patients) resulted in marked apoptosis and necrosis of both cell types. **(B)** CXCR1/2 blockade showed significant reduction in inflammatory cytokines/chemokines, including CXCL8/IL-8, IL-6, IL-23, and CCL20. However, GM-CSF and IL-17 was increased. Bar graphs demonstrate cytokines production after LPS stimulation **(C)** To investigate the effect of CXCR1/2 blockade on reactive oxygen species (ROS) production, cells were stimulated with *E. coli* for 18 h, and production of ROS was measured. Bar graph illustrates significant reduction in ROS after CXCR1/2 blockade. **(D)** Immunofluorescence confirmed significant reduction in ROS which started at 3 h completely depleted at 18 h (magnification: 20×) (*n* = 15). **(E)** MFI of ROS was calculated at different time point after CXCR1/2 blockade and shown in bar graph.

First, to explore whether neutrophil’s inflammatory mediators induce cell death, HepG2 and HepG2.2.15 cells were cultured with supernatant obtained from resting and LPS-activated neutrophils isolated from ACLF patients. We observed significant apoptosis and necrosis of both HepG2 and HepG2.2.15 cells (Figure 6A), which suggest that neutrophil’s inflammatory mediators (CXCL8/IL-8, IL-6, IL-17, IL-23, GM-CSF, CCL-20, and ROS) are conductive toward cell death. Further, to find out that CXCR1/2 signaling had any effect on the production of neutrophil’s inflammatory mediators; CXCR1/2 blockade was performed. Blockade of CXCR1/2 resulted in a significant reduction in inflammatory mediators, including CXCL8/IL-8 (a well-known CXCR1/2 ligand), IL-6, IL-23, and CCL20, in patient groups as well as HC which in turn prevented cell death. However, GM-CSF and IL-17 production was enhanced (Figure 6B). Notably, substantial reduction in ROS was also seen after CXCR1/2 blockade (Figure 6C). Further, to investigate the time point when reduction in ROS was started after CXCR1/2 blockade, ROS production was measured at different time points by immunofluorescence microscopy. We observed significant decline in ROS at 3 h which continued till 6 h and was completely inhibited at 18 h (Figure 6D). MFI of ROS was calculated at different time points which also indicated a significant reduction of ROS at 3 h and absolutely diminished at 18 h (Figure 6E). Therefore, our data strongly suggest that CXCR1/2 not only induce contact-dependent cell death but also encourage cell death through contact-independent mechanism by increasing the production of inflammatory mediators. Consequently, blockade of CXCR1/2 not only helps in preventing contact-dependent cell death but also inhibits contact-independent cell death.

## Discussion

**Figure 7:**
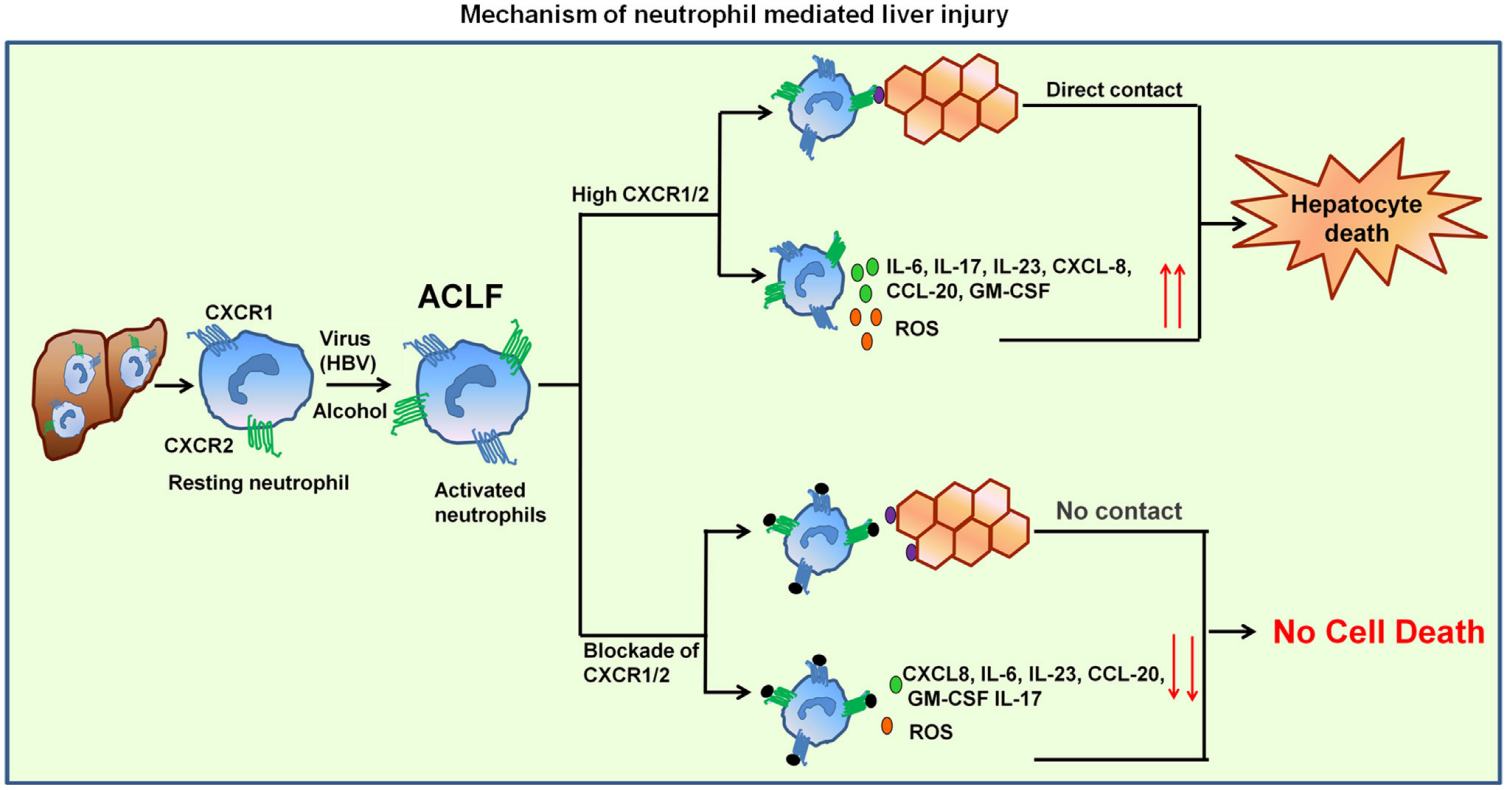
**Mechanism of neutrophil-mediated liver damage in ACLF**. Induction of neutrophils by virus (HBV) or alcohol leads to hyper activation resulting in increased CXCR1/2. Neutrophil then migrate toward liver where they contact to hepatocytes in CXCR1/2-dependent mechanism and induce cell death. High CXCR1/2 increases the production of reactive oxygen species (ROS) and pro-inflammatory cytokines which leads to protracted inflammation, tissue destruction, and ultimately organ dysfunction. Blockade of CXCR1/2 not only reduced contact-dependent cell death but also abridged production of ROS and pro-inflammatory mediators, thereby decreasing the inflammatory response and liver damage.

Neutrophils seem to play a crucial role in the pathogenesis of viral (24, 26) and bacterial infections (27, 28), alcoholic hepatitis (29, 30), ALF (31), and sepsis (7, 32). Results of the present study clearly indicate that neutrophils over-expressing CXCR1 and CXCR2 contribute to hepatocyte death by direct contact as well as through inflammatory mediators by inducing early apoptosis and necrosis, suggesting this might be an underlying mechanism operating during the impairment of liver function in ACLF. Importantly, CXCR1/2 blockade inhibited cell death mediated by both contact-dependent as well as -independent mechanism. Graphical abstract represent the mechanism of neutrophil-mediated liver injury (Figure 7).

A recent study has revealed that when increasing number of neutrophils from healthy control were incubated with HepG2 cells; they caused cytotoxicity by a cell contact-dependent manner. However, the study does not discriminate if cell death was due to apoptosis or necrosis or by both the ways. Moreover, the study also lacks which neutrophil molecule is responsible for this cytotoxicity which could be targeted for therapeutic purpose (11). Our work for the first time established that neutrophil’s CXCR1/2 is important for mediating both apoptotic and necrotic cell death. Noticeably, blockade of CXCR1/2 with SCH 527123 antagonist significantly reduced cell death, which strongly signifies CXCR1/2 as a potential therapeutic target. Studies performed on murine models of acetaminophen-induced liver injury also showed that CXCR2-mediated infiltration of neutrophils amplifies liver injury, contributes to systemic inflammation, and promotes further organ injury, since blockade of CXCR2 significantly reduces hepatotoxicity (33). Moreover, recent study also showed that blocking of CXCR1/2 receptors with pepducins increases survival of a murine model of alcoholic liver disease, reverts liver inflammation and steatosis (34). Most of the studies are performed on animal models; however, we have checked the effect of CXCR1/2 blockade on human neutrophils.

Importantly, studies have demonstrated critical role of CXCR1 and CXCR2 in tumor growth and metastasis and serves as the key target for the development of novel therapies. It has been reported that targeting CXCR1 and CXCR2 using orally active small-molecule antagonist, SCH 527123, potentially inhibits human colon cancer liver metastasis (16) and also sensitizes cells to oxaliplatin (17). Recently, CXCR1 and CXCR2 antagonist are used to treat human melanoma (18), lung (19), breast (20), and pancreatic cancer (21) growth by inhibiting tumor cell proliferation and suppression of angiogenesis and metastasis. Therefore, it is significant considering the upregulated expression of CXCR1 and CXCR2 in ACLF patients which may offer a crucial target for the development of new therapies. However targeting CXCR1/2 for therapeutic interventions requires validation studies on large cohort of ACLF patients.

Data from recent study also demonstrated circulating neutrophil dysfunction in patients with cirrhosis, which could predict the development of infection, organ dysfunction, and associated with 90-day and 1-year mortality (31). In our study, multivariate analysis also revealed ANC (>73.5) and CXCL8/IL-8 (>27) as a predictor of mortality. However, ANC had higher sensitivity and specificity in predicting mortality. Activated neutrophils release ROS, which have bactericidal properties. However, high amount of ROS may also harm neighboring cells and promote further tissue damage. In ACLF, neutrophils devastating capacity to produce large amount of ROS resulted in tissue injury and cell death. Studies have shown that high ROS induces oxidative stress in hepatocytes resulting in cell death. However, there is no study indicating the possible mechanism to control the production of ROS. Our data revealed that blocking of CXCR1/2 significantly reduce ROS production. It is well known that ACLF patients are prone to infection and sepsis develops rather early (35, 36), impaired NPA in these patients may add to disease severity since neutrophils would not be able to clear infection. In healthy adults, neutrophils are short lived in circulation and exist in a resting state, which ensures that their toxic intracellular contents are not accidentally released to damage host tissue. However, in diseased state, neutrophils are highly activated and have a prolonged survival, which is supported by a cytokine milieu G-CSF, GM-CSF, IL-6, TNF-α, and IFN-γ. We observed elevated levels of GM-CSF and IL-6 in ACLF which may maintain extended survival of neutrophils. Interestingly, we also found that neutrophil produce high amount of IL-23. In fact, previous studies considered dendritic cells and macrophages as the main source of IL-23. However, we showed that neutrophils are high producers of IL-23 along with IL-17, CXCL8/IL-8, and CCL-20. IL-23 has a major role in generation of pathogenic Th17 cells (37, 38), which have been involved in viral hepatitis caused by HBV (39, 40), HCV (41), as well as autoimmune arthritis (42) and other chronic diseases (43). Another important observation emerging from this study was the production of CCL-20 by neutrophils, a known ligand for CCR-6 which is mainly present on Th17 cells. CCL-20 is the most crucial cytokine for Th17 cells migration to the site of injury. CXCL8/IL-8 is also a key chemokine, which is not only involved in the generation hyper-inflammatory responses but also triggers leukocyte recruitment to the infection sites. The amount of CXCL8/IL-8 produced at the site of inflammation determines in fact, the magnitude of leukocyte infiltration and activation. Our results demonstrate that CXCR1/2 blockade with SCH 527123 antagonist inhibit the production of inflammatory cytokines/chemokines and reduce cell death.

In conclusion, our study suggests that neutrophils play a central role in pathogenesis of ACLF. Regardless of the specific etiology, ACLF patients have an increased number of neutrophils which have high expressions of liver homing receptors, CXCR1 and CXCR2, inducing hepatocyte death by early apoptosis and necrosis. Blockade of CXCR1/2 with SCH 527123 antagonist significantly abrogate cell death; therefore, pharmacological neutralization of CXCR1/2 provides a valid target for new therapeutic interventions which may help in management of ACLF.

## Ethics Statement

Study protocol was approved by the ethical committee of Institute of Liver and Biliary Sciences (Ethical approval number: F.1/8/22/AC/Admn/ILBS/2010/09-07-2011). Study was performed in accordance with the principles of Helsinki Declaration. Written informed consent was received from all the participants or their family members; in case the patient was unable give consent.

## Author Contributions

AK: collection of patient material, data collection, acquisition, analysis and interpretation, statistical analysis, and manuscript writing. SS, NT, CG, and PR: revision of the manuscript. PR: characterization of neutrophils. AR: analysis of IHC data. SS, NT, and AK: study concept and design. SS: selection of samples and critical revision of manuscript done for important intellectual content.

## Conflict of Interest Statement

The authors declare that the research was conducted in the absence of any commercial or financial relationships that could be construed as a potential conflict of interest.

## Abbreviations

ACLF, acute-on chronic liver failure; CHB, chronic hepatitis B; ALT, alanine amino transferase; ROS, reactive oxygen species; ANC, absolute neutrophil count; NPA, neutrophil phagocytic activity; MELD, model of end-stage liver disease; CTP, child turcotte pugh; INR, international normalized ratio; ROC, receiver operating curve.

## References

[1] Jalan R Gines P Olson JC Mookerjee RP Moreau R Garcia-Tsao G Acute-on chronic liver failure. J Hepatol (2012) 57:1336–48. 10.1016/j.jhep.2012.06.026 22750750

[2] Chan AC Fan ST Lo CM Liu CL Chan SC Ng KK Liver transplantation for acute-on-chronic liver failure. Hepatol Int (2009) 3:571–81. 10.1007/s12072-009-9148-8 19680733 PMC2790588

[3] Khanam A Trehanpati N Garg V Kumar C Garg H Sharma BC Altered frequencies of dendritic cells and IFN-gamma-secreting T cells with granulocyte colony-stimulating factor (G-CSF) therapy in acute-on-chronic liver failure. Liver Int (2014) 34:505–13. 10.1111/liv.12415 24754047

[4] Lee WL Harrison RE Grinstein S Phagocytosis by neutrophils. Microbes Infect (2003) 5:1299–306. 10.1016/j.micinf.2003.09.014 14613773

[5] Sareila O Kelkka T Pizzolla A Hultqvist M Holmdahl R. NOX2 complex-derived ROS as immune regulators. Antioxid Redox Signal (2011) 15:2197–208. 10.1089/ars.2010.3635 20919938

[6] Ramaiah SK Jaeschke H. Role of neutrophils in the pathogenesis of acute inflammatory liver injury. Toxicol Pathol (2007) 35:757–66. 10.1080/01926230701584163 17943649

[7] Brown KA Brain SD Pearson JD Edgeworth JD Lewis SM Treacher DF. Neutrophils in development of multiple organ failure in sepsis. Lancet (2006) 368:157–69. 10.1016/S0140-6736(06)69005-3 16829300

[8] Taylor NJ Nishtala A Manakkat Vijay GK Abeles RD Auzinger G Bernal W Circulating neutrophil dysfunction in acute liver failure. Hepatology (2013) 57:1142–52. 10.1002/hep.26102 23079896

[9] Fiuza C Salcedo M Clemente G Tellado JM. In vivo neutrophil dysfunction in cirrhotic patients with advanced liver disease. J Infect Dis (2000) 182:526–33. 10.1086/315742 10915084

[10] Bautista AP. Neutrophilic infiltration in alcoholic hepatitis. Alcohol (2002) 27:17–21. 10.1016/S0741-8329(02)00206-9 12062632

[11] Marques PE Amaral SS Pires DA Nogueira LL Soriani FM Lima BH Chemokines and mitochondrial products activate neutrophils to amplify organ injury during mouse acute liver failure. Hepatology (2012) 56:1971–82. 10.1002/hep.25801 22532075

[12] Holmes WE Lee J Kuang WJ Rice GC Wood WI. Structure and functional expression of a human interleukin-8 receptor. Science (1991) 253:1278–80. 10.1126/science.1840701 1840701

[13] Murphy PM Tiffany HL. Cloning of complementary DNA encoding a functional human interleukin-8 receptor. Science (1991) 253:1280–3. 10.1126/science.1891716 1891716

[14] Kobayashi Y. The role of chemokines in neutrophil biology. Front Biosci (2008) 13:2400–7. 10.2741/2853 17981721

[15] Kuboki S Shin T Huber N Eismann T Galloway E Schuster R Hepatocyte signaling through CXC chemokine receptor-2 is detrimental to liver recovery after ischemia/reperfusion in mice. Hepatology (2008) 48:1213–23. 10.1002/hep.22471 18688883 PMC2695827

[16] Varney ML Singh S Li A Mayer-Ezell R Bond R Singh RK. Small molecule antagonists for CXCR2 and CXCR1 inhibit human colon cancer liver metastases. Cancer Lett (2011) 300:180–8. 10.1016/j.canlet.2010.10.004 21035946 PMC2994987

[17] Ning Y Labonte MJ Zhang W Bohanes PO Gerger A Yang D The CXCR2 antagonist, SCH-527123, shows antitumor activity and sensitizes cells to oxaliplatin in preclinical colon cancer models. Mol Cancer Ther (2012) 11:1353–64. 10.1158/1535-7163.MCT-11-0915 22391039

[18] Singh S Sadanandam A Nannuru KC Varney ML Mayer-Ezell R Bond R Small-molecule antagonists for CXCR2 and CXCR1 inhibit human melanoma growth by decreasing tumor cell proliferation, survival, and angiogenesis. Clin Cancer Res (2009) 15:2380–6. 10.1158/1078-0432.CCR-08-2387 19293256 PMC4232212

[19] Khan MN Wang B Wei J Zhang Y Li Q Luan X CXCR1/2 antagonism with CXCL8/interleukin-8 analogue CXCL8(3-72)K11R/G31P restricts lung cancer growth by inhibiting tumor cell proliferation and suppressing angiogenesis. Oncotarget (2015) 6:21315–27. 10.18632/oncotarget.4066 26087179 PMC4673267

[20] Singh JK Farnie G Bundred NJ Simoes BM Shergill A Landberg G Targeting CXCR1/2 significantly reduces breast cancer stem cell activity and increases the efficacy of inhibiting HER2 via HER2-dependent and -independent mechanisms. Clin Cancer Res (2013) 19:643–56. 10.1158/1078-0432.CCR-12-1063 23149820 PMC4868141

[21] Hertzer KM Donald GW Hines OJ CXCR2: a target for pancreatic cancer treatment? Expert Opin Ther Targets (2013) 17:667–80. 10.1517/14728222.2013.772137 23425074 PMC3686651

[22] Sarin SK Kumar A Almeida JA Chawla YK Fan ST Garg H Acute-on-chronic liver failure: consensus recommendations of the Asian Pacific Association for the study of the liver (APASL). Hepatol Int (2009) 3:269–82. 10.1007/s12072-008-9106-x 19669378 PMC2712314

[23] Sarin SK Kedarisetty CK Abbas Z Amarapurkar D Bihari C Chan AC Acute-on-chronic liver failure: consensus recommendations of the Asian Pacific Association for the study of the liver (APASL) 2014. Hepatol Int (2014) 8:453–71. 10.1007/s12072-014-9580-2 26202751

[24] Alvarez-Uria G Day JN Nasir AJ Russell SK Vilar FJ. Reduction in neutrophil count during hepatitis C treatment: drug toxicity or predictor of good response? Dig Dis Sci (2010) 55:2058–62. 10.1007/s10620-009-0969-z 19757045

[25] Russo RC Garcia CC Teixeira MM Amaral FA. The CXCL8/IL-8 chemokine family and its receptors in inflammatory diseases. Expert Rev Clin Immunol (2014) 10:593–619. 10.1586/1744666X.2014.894886 24678812

[26] Takai S Kimura K Nagaki M Satake S Kakimi K Moriwaki H. Blockade of neutrophil elastase attenuates severe liver injury in hepatitis B transgenic mice. J Virol (2005) 79:15142–50. 10.1128/JVI.79.24.15142-15150.2005 16306586 PMC1315990

[27] Rigby KM Deleo FR. Neutrophils in innate host defense against *Staphylococcus aureus* infections. Semin Immunopathol (2012) 34:237–59. 10.1007/s00281-011-0295-3 22080185 PMC3271231

[28] Kolaczkowska E Kubes P. Neutrophil recruitment and function in health and inflammation. Nat Rev Immunol (2013) 13:159–75. 10.1038/nri3399 23435331

[29] Gao B Bataller R. Alcoholic liver disease: pathogenesis and new therapeutic targets. Gastroenterology (2011) 141:1572–85. 10.1053/j.gastro.2011.09.002 21920463 PMC3214974

[30] Bertola A Park O Gao B. Chronic plus binge ethanol feeding synergistically induces neutrophil infiltration and liver injury in mice: a critical role for E-selectin. Hepatology (2013) 58:1814–23. 10.1002/hep.26419 23532958 PMC3726575

[31] Taylor NJ Manakkat Vijay GK Abeles RD Auzinger G Bernal W Ma Y The severity of circulating neutrophil dysfunction in patients with cirrhosis is associated with 90-day and 1-year mortality. Aliment Pharmacol Ther (2014) 40:705–15. 10.1111/apt.12886 25060167

[32] Reddy RC Standiford TJ. Effects of sepsis on neutrophil chemotaxis. Curr Opin Hematol (2010) 17:18–24. 10.1097/MOH.0b013e32833338f3 19864946

[33] Liu ZX Han D Gunawan B Kaplowitz N. Neutrophil depletion protects against murine acetaminophen hepatotoxicity. Hepatology (2006) 43:1220–30. 10.1002/hep.21175 16729305

[34] Wieser V Adolph TE Enrich B Kuliopulos A Kaser A Tilg H Reversal of murine alcoholic steatohepatitis by pepducin-based functional blockade of interleukin-8 receptors. Gut (2017) 66:930–8. 10.1136/gutjnl-2015-310344 26858343 PMC5531226

[35] Yu Z Li F Zeng Z Huang Z Fan Z Jin Y Prevalence and clinical significance of *Cryptosporidium* infection in patients with hepatitis B virus-associated acute-on-chronic liver failure. Int J Infect Dis (2011) 15:e845–8. 10.1016/j.ijid.2011.08.006 21992928

[36] Lin KH Liu JW Chen CL Wang SH Lin CC Liu YW Impacts of pretransplant infections on clinical outcomes of patients with acute-on-chronic liver failure who received living-donor liver transplantation. PLoS One (2013) 8:e72893. 10.1371/journal.pone.0072893 24023787 PMC3759387

[37] Lee Y Awasthi A Yosef N Quintana FJ Xiao S Peters A Induction and molecular signature of pathogenic TH17 cells. Nat Immunol (2012) 13:991–9. 10.1038/ni.2416 22961052 PMC3459594

[38] Wu C Yosef N Thalhamer T Zhu C Xiao S Kishi Y Induction of pathogenic TH17 cells by inducible salt-sensing kinase SGK1. Nature (2013) 496:513–7. 10.1038/nature11984 23467085 PMC3637879

[39] Sun HQ Zhang JY Zhang H Zou ZS Wang FS Jia JH. Increased Th17 cells contribute to disease progression in patients with HBV-associated liver cirrhosis. J Viral Hepat (2012) 19:396–403. 10.1111/j.1365-2893.2011.01561.x 22571901

[40] Yang B Wang Y Zhao C Yan W Che H Shen C Increased Th17 cells and interleukin-17 contribute to immune activation and disease aggravation in patients with chronic hepatitis B virus infection. Immunol Lett (2013) 149:41–9. 10.1016/j.imlet.2012.12.001 23237940

[41] Chang Q Wang YK Zhao Q Wang CZ Hu YZ Wu BY. Th17 cells are increased with severity of liver inflammation in patients with chronic hepatitis C. J Gastroenterol Hepatol (2012) 27:273–8. 10.1111/j.1440-1746.2011.06782.x 21592230

[42] Torres T Filipe P. [Interleukin-17 as a therapeutic target in psoriasis]. Acta Med Port (2014) 27:252–8. 10.20344/amp.4777 24813495

[43] Yu X Guo R Ming D Su M Lin C Deng Y Ratios of regulatory T cells/T-helper 17 cells and transforming growth factor-beta1/interleukin-17 to be associated with the development of hepatitis B virus-associated liver cirrhosis. J Gastroenterol Hepatol (2014) 29:1065–72. 10.1111/jgh.12459 24236690

