# Supplementary material for: Blockade of Neutrophil’s Chemokine Receptors CXCR1/2 Abrogate Liver Damage in Acute-on-Chronic Liver Failure
Source: Front Immunol. 2017 Apr 24;8:464. doi: 10.3389/fimmu.2017.00464 (PMC5401894; doi:10.3389/fimmu.2017.00464)
Supplement: Supplementary file 1 [file image_1.pdf]

SUPPLEMENTARY FIGURE 1

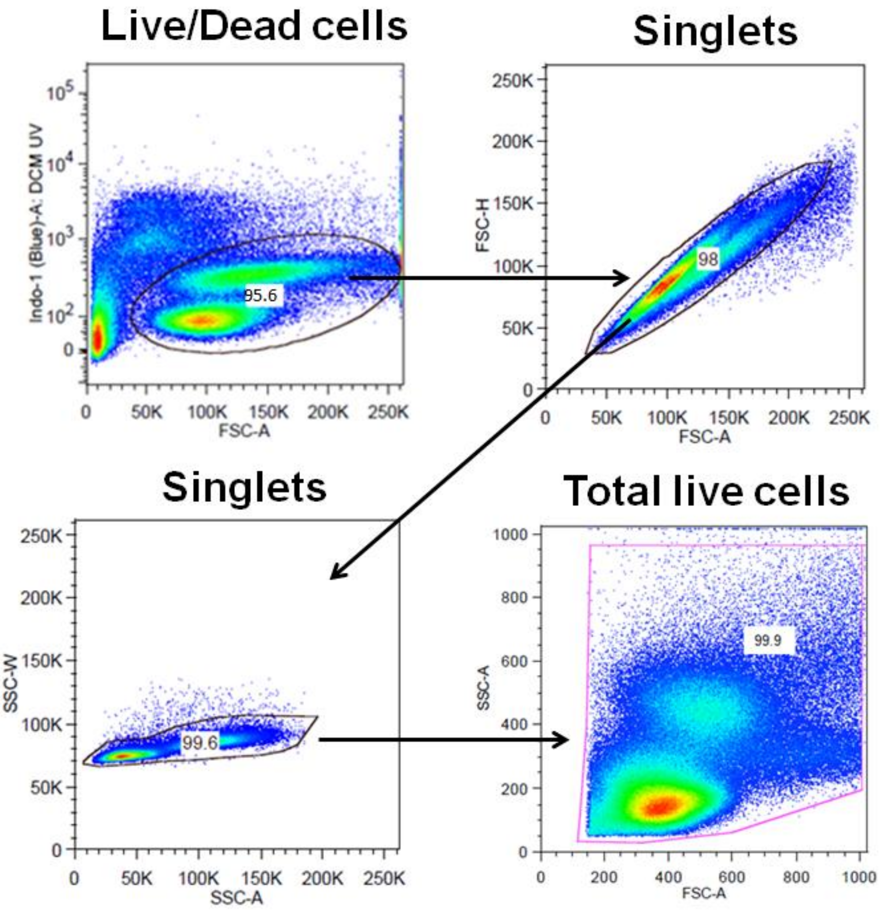

Supplementary Figure 1 Gating strategy of neutrophils for flow cytometry analysis. Gating was based on the following strategy: FSC-A vs. Indo-1(Blue)-A:DCM UV for the selection of live cells, FSC-A vs. FSC-H for singlets, SSC-A vs. SSC-W for the better resolution of multiplets, FSC vs. SSC for gating the cells on the basis of size and granularity respectively. Neutrophils were then analyzed on the basis of CD11b and CD16 positivity.
