# Supplementary material for: Blockade of Neutrophil’s Chemokine Receptors CXCR1/2 Abrogate Liver Damage in Acute-on-Chronic Liver Failure
Source: Front Immunol. 2017 Apr 24;8:464. doi: 10.3389/fimmu.2017.00464 (PMC5401894; doi:10.3389/fimmu.2017.00464)
Supplement: Supplementary file 2 [file image_2.pdf]

SUPPLEMENTARY FIGURE 2

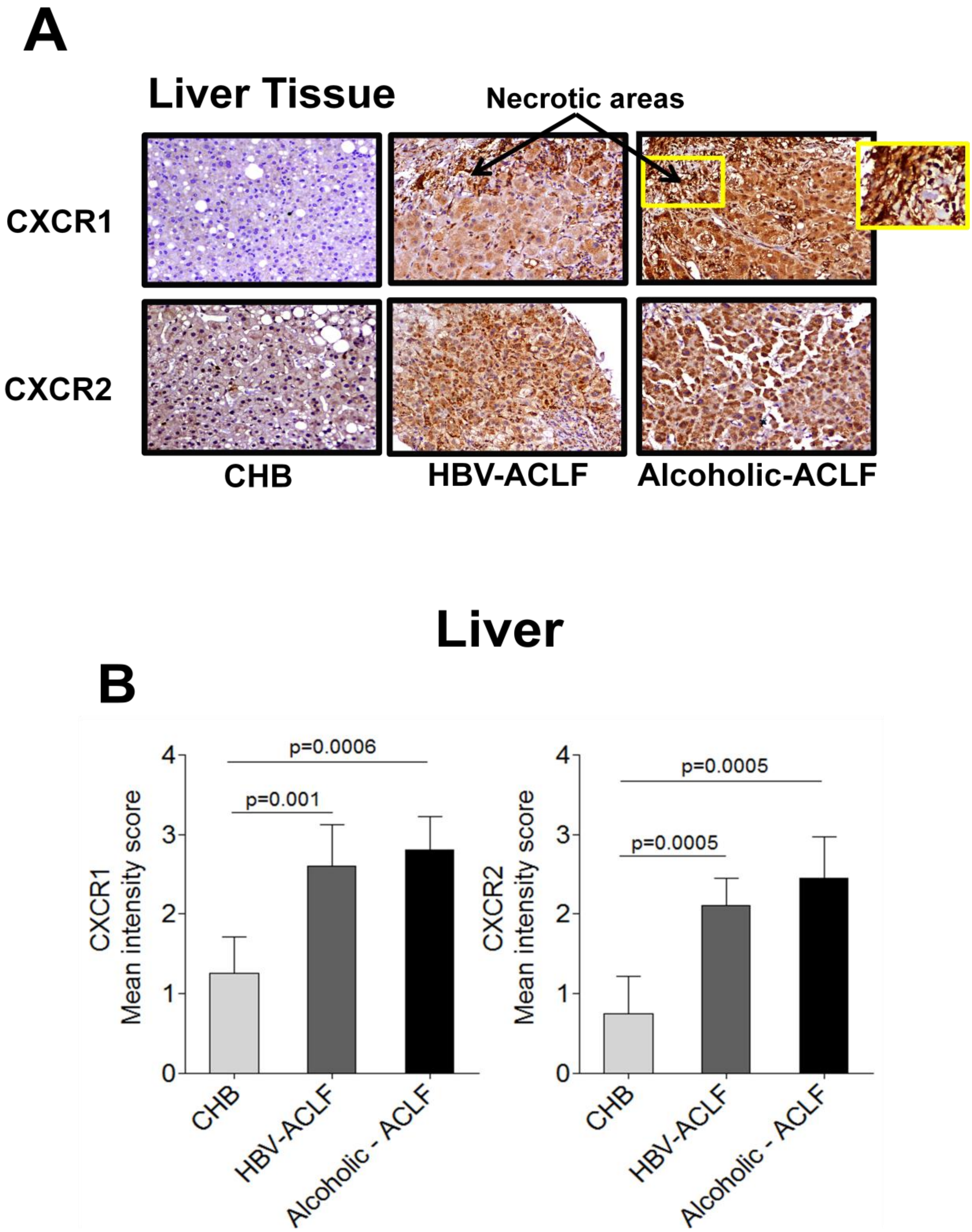

Supplementary Figure 2 Increased CXCR1 and CXCR2 expression in the liver tissue of ACLF. (A)Immunohistochemistry (IHC) data illustrates high CXCR1 and CXCR2 staining in ACLF, more intense near necrotic areas of liver (magnification: 20X). (B)IHC data scoring

was done by giving a numerical value on a scale of 0, 1, 2 and 3 for none, light, medium and dark respectively. Higher CXCR1 and CXCR2 mean intensity score was found in ACLF groups than CHB.
