# Supplementary material for: Blockade of Neutrophil’s Chemokine Receptors CXCR1/2 Abrogate Liver Damage in Acute-on-Chronic Liver Failure
Source: Front Immunol. 2017 Apr 24;8:464. doi: 10.3389/fimmu.2017.00464 (PMC5401894; doi:10.3389/fimmu.2017.00464)
Supplement: Supplementary file 3 [file image_3.pdf]

SUPPLEMENTARY FIGURE 3

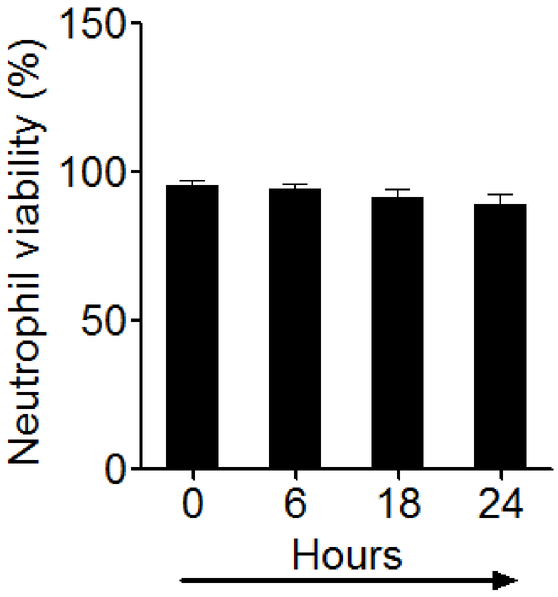

Supplementary Figure 3 Viability of neutrophils. Neutrophils viability was checked at different time point to find out whether they would survive throughout the incubation period in neutrophils and HepG2/HepG2.2.15 co-culture. Result showed that 90% neutrophils survived till 24 hrs.
